# Capillary Electrophoresis Electrospray Ionization Mass Spectrometry Reveals Metabolic Perturbations During Nematode Infection in *Drosophila melanogaster*

**DOI:** 10.3390/molecules30092023

**Published:** 2025-05-01

**Authors:** Yayra T. Tuani, Navid J. Ayon, Rosemary M. Onjiko, Sam B. Choi, Shruti Yadav, Ioannis Eleftherianos, Peter Nemes

**Affiliations:** 1 Department of Chemistry & Biochemistry, University of Maryland, College Park, MD 20742, USA; ytuani@umd.edu (Y.T.T.); njayon@umd.edu (N.J.A.); msonjiko@gwmail.gwu.edu (R.M.O.); choib19@gwmail.gwu.edu (S.B.C.); 2 Department of Chemistry, The George Washington University, Washington, DC 20052, USA; 3 Department of Biological Sciences, The George Washington University, Washington, DC 20052, USA; shrutiyadav@gwmail.gwu.edu (S.Y.); ioannise@gwu.edu (I.E.)

**Keywords:** metabolomics, *Steinernema carpocapsae*, *Xenorhabdus nematophila*, nematode, parasitism, symbiosis

## Abstract

*Drosophila melanogaster* is broadly used to model host–pathogen interactions. Entomopathogenic nematodes are excellent research tools for dissecting the molecular and functional basis of parasitism and the host’s anti-parasitic response. In this work, we used discovery metabolomics to explore the differences in the metabolome composition of wild type *D. melanogaster* larvae that were infected with symbiotic nematodes (*Steinernema carpocapsae* carrying *Xenorhabdus nematophila* mutualistic bacteria) or axenic nematodes (*S. carpocapsae* lacking their bacterial partners). Benefiting from their high separation power, sensitivity, and compatibility with low amounts of the starting metabolome, we leveraged microanalytical capillary electrophoresis electrospray ionization mass spectrometry (CE-ESI-MS) to profile the small (<500 Da) polar portion of the metabolome among these experimental treatments. We detected and quantified 122 different small molecules, of which 50 were identified with high confidence. Supervised multivariate analysis revealed that the infection was paralleled with changes in amino acid biosynthesis (arginine, phenylalanine, tryptophan, and tyrosine), metabolism (alanine, arginine, aspartate, glutamate, glycine, proline, serine, and threonine), and classical signalling (aspartate, γ-aminobutyrate, glutamate, and pyridoxine). This study demonstrates the ability of high-sensitivity CE-ESI-MS to uncover metabolic perturbations during infection. The results from the metadata may facilitate the design of targeted studies to explore small biomolecules and their functions during host–pathogen interaction.

## 1. Introduction

Contrary to the classical hypothesis, pathogens do not always lead to host disease but can lead to an ecological synergy where both host and pathogens benefit from each other in a process known as mutualism [1]. Studying host–pathogen interaction helps to understand the biochemical basis of pathogenesis, including cellular signalling and communication, immune regulation, and cellular response. These insights, in turn, can aid in identifying new therapeutic targets and anti-infectious drug development [2]. The metabolome, the comprehensive suite of metabolites produced downstream of transcription and translation, provides an efficient and dynamic measure of the molecular state of the system. Metabolomics is a nascent field in the study of host–pathogen interactions. Some studies reported altered metabolic pathways during host–pathogen interaction, such as glycolysis, fatty acid production, and amino acid biosynthesis [3]; however, a comprehensive metabolomics analysis during such encounters has yet to be performed. Metabolome profiling has been leveraged to reflect on upstream protein regulation [4].

*Drosophila melanogaster*, the common fruit fly, combines many advantages as a model organism for modelling host–pathogen interactions [5], including studies in bioenergetics and nutrient metabolism [6]. It has a low maintenance cost, reproduces quickly, and has a well-characterized genome that is amenable to modification via diverse genetic tools [7]. Further, *D. melanogaster* can be genetically reared germfree, allowing for investigations on the intricate relationship between the host and pathogen [8]. The generation of biological model systems of these features in other species, especially mammalian ones such as rodents, is considerably more expensive and can be technically challenging. Apart from the idiosyncratic differences, *D. melanogaster* is genetically sufficiently similar to its mammalian counterparts to help explore evolutionary conserved interaction pathways. The fly has been used in studies on immune cascades, signal transduction, and transcriptional regulation (see the reviews in Ref. [9]). *D. melanogaster* can be readily infected with a variety of pathogens, and molecular and genetic tools are available to probe effector molecules, pathways, and virulence factors [9]. Intriguingly, *D. melanogaster* lacks an adaptive immune response and solely relies on innate immunity to fight off pathogenic invasion, primarily through the production of antimicrobial peptides, anti-pathogenic metabolites, and antiviral factors [10,11]. These features have rendered the fly a suitable model for studying host–pathogen interactions via an in-depth exploration of cellular processes and the biosynthesis of endogenous molecules.

Several studies have adapted *D. melanogaster* to identify the processes and host factors involved in cellular entry, controlling or resisting bacterial infection, and other factors undergoing dysregulation during bacterial infection [12,13,14,15]. Mutants and transgenic flies have been used to study the roles of genes in response to infection with diverse pathogens through varying immune response pathways [16]. A phenotypic study using a machine learning-based image analysis platform (e.g., HRMAn [17]) recently helped to recognize the cellular defence responses of *Toxoplasma gondii* and *Salmonella enterica* Typhimurium [17]. Factors leading to fly resistance to *Listeria monocytogenes* were investigated using genetic screens, and genes controlling immunity and pathogenesis were found [18]. Moreover, *D. melanogaster* has helped recapitulate the phenotypes of certain human pathogens, such as the Zika virus, *Mycobacterium marinum*, *Listeria monocyotgenes,* and *Candida albicans* [14,19].

The entomopathogenic (or insect pathogenic) nematode *Steinernema carpocapsae,* along with its mutualistic bacterial partner, *Xenorhabdus nematophila,* are a good model for nematode–bacterium symbiosis. For example, microbial mutualism and pathogenesis can be explored simultaneously in insect–nematode–bacteria in vivo studies [20,21,22]. The invasion of entomopathogenic nematodes into the insect hemocoel (insect body cavity) is followed by the release of the symbiotic bacteria, which produce virulence factors and toxins that degrade tissues, leading to insect death. The nematodes and their bacteria then multiply and complete their life cycle in the insect corpse before the new generation of parasites appears and reassociates with the bacteria. Finally, the nematode–bacteria complexes exit the dead host to target and infect new insect hosts [23]. While genomic approaches to studying host–pathogen interactions using *D. melanogaster* are well-established, ‘omics technologies such as metabolomics and proteomics have only recently started to be used to identify the molecular mechanisms responsible for these interactions [24,25,26,27]. The metabolome can respond rapidly and dynamically to effectors through molecular mechanisms regulated by complex interconnected pathways downstream of transcription and translation. It is, therefore, an emerging target for future host–pathogen studies.

High-resolution tandem mass spectrometry (MS^2^) can sensitively and accurately profile complex metabolomes. MS can also be used in quantitative analysis to endogenous concentrations [24]. Front-end separations aid in metabolome coverage by removing interferences during co-elution, ionization, and signal detection/quantification. Gas and liquid chromatography (GC, LC) usually call for derivatization and need large amounts of samples, typically ~5–50 µL in volume. Without derivatization, capillary electrophoresis (CE) can efficiently separate ionic species, even from ~10–100 nL samples. CE-MS has enabled the detection of 1692 compounds in *Bacillus subtilis* cells, including 150 that could be identified [25]. We and others have developed custom-built high-sensitivity CE-MS platforms to characterize single-cell metabolomes. Ca. 80–150 metabolites were identified in neurons dissected from *Aplysia californica* and the rat [26,27,28] and identified blastomeres in 2-cell zebrafish and 8- to 16-cell *Xenopus laevis* embryos, uncovering a cell-to-cell metabolic heterogeneity capable of altering normal cell fate decisions [29,30,31,32,33]. Metabolic perturbations can direct immune response, e.g., via T-cell signalling molecules [34]. Elevated levels of *D*-amino acids and classical neurotransmitter indicated disrupted cell-to-cell signalling within the islets during diabetes [35].

Here, we leverage high-sensitivity microanalytical CE-MS to explore the metabolic differences between *D. melanogaster* larvae infected with either symbiotic (Sym) or axenic (Axe) *S. carpocapsae* nematodes carrying or lacking their mutualistic bacteria *X. nematophila*, respectively, and uninfected control larvae (Ctrl). We hypothesized that *D. melanogaster* would exhibit differences in its cellular biochemistry during the encounter with the nematode, reflected by shifts in the exhibited metabolome. Microanalytical CE-MS was chosen to separate and quantify ~120 of the small, polar, and charged compounds, 50 of which were identified against an in-house-built CE-ESI-MS metabolome database using the same analytical platform used in this study [29]. Amino acids, osmolytes, energy carriers, and classical signalling molecules were the foci of follow-up supervised and unsupervised multivariate data analyses to define the metabolome profiles of the Axe, Sym, and Ctrl nematode infections.

## 2. Results and Discussion

**Figure 1:**
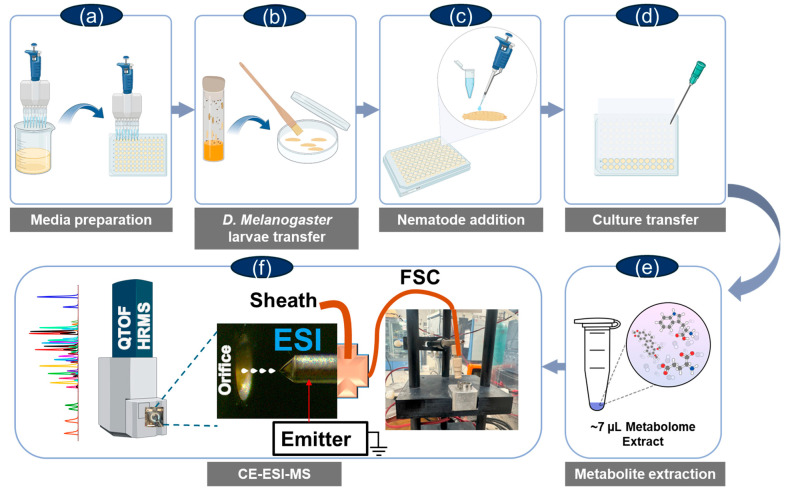
Our experimental plan to profile host–pathogen interactions involving *Drosophila melanogaster,* the entomopathogenic nematode *Steinernema carpocapsae* carrying the mutualistic bacteria *Xenorhabdus nematophila* (symbiotic worms), and the axenic *S. carpocapsae* nematodes (free of *X. nematophila* bacteria). (**a**) Agarose gel was added to the wells of a 96 well plate. (**b**) Third instar *D. melanogaster* larvae (Oregon strain) were transferred onto a Whatman filter paper in a Petri dish using a fine soft bristle paintbrush. (**c**) A small drop of sterile water was used to wash the larvae. (**d**) Sterile distilled water containing symbiotic or axenic surface-sterilized *S. carpocapsae* nematodes and a single *D. melanogaster* larva were added to each well of the microtiter plate. (**e**) The small, polar portion of the metabolomes was micro-extracted using acidified organic solvents. (**f**) The sample was analyzed using a validated, custom-built CE-ESI platform on a quadrupole time-of-flight (QTOF) high-resolution mass spectrometer (HRMS).

We employed microanalytical CE-ESI-MS to profile the polar, small metabolites (<500 Da) during the host–pathogen interactions involving *D. melanogaster* larvae. The studies were split into the Ctrl, Sym, and Axe groups, as described above. Figure 1 shows the combination used in our workflow, encompassing nematode infection of *D. melanogaster* larvae and metabolome extraction and CE-ESI-MS measurement. *D. melanogaster* larvae were uninfected or they were infected with either symbiotic or axenic *S. carpocapsae* nematodes. All larvae were collected 78 h post infection, which corresponds to the median lethal dose [36], and samples were prepared for metabolomics analysis. Upon metabolite extraction, ~10 nL of each metabolite extract was measured on our validated microanalytical CE-ESI-MS platform [29,30,37].

**Figure 2:**
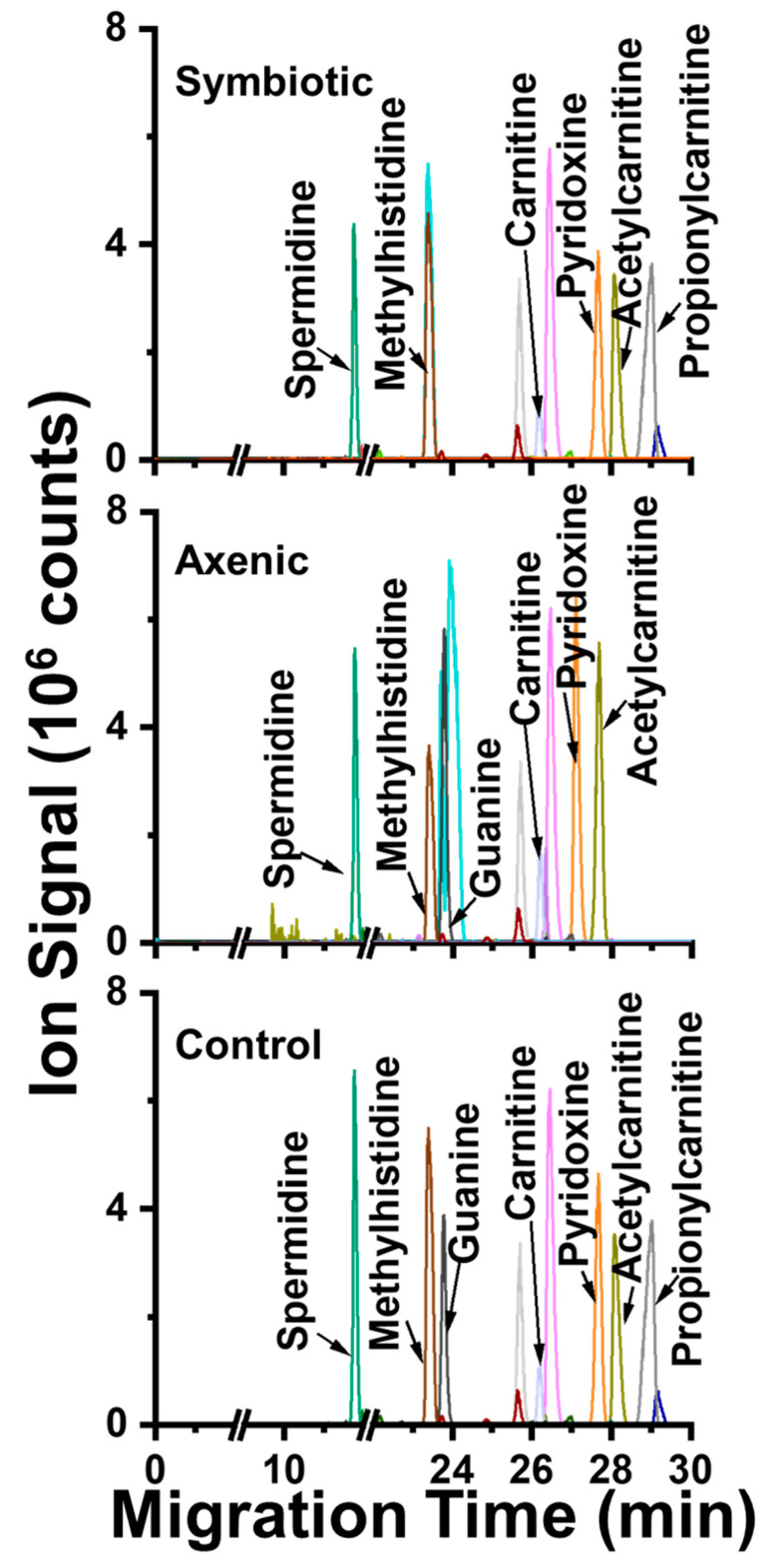
Metabolome profiling in *D. melanogaster* larvae infected by axenic or symbiotic *Steinernema carpocapsae* entomopathogenic nematodes. The small and polar metabolomes were extracted in 50% acidified methanol and analyzed in a validated CE-ESI-MS platform. A total of 122 molecular features (unique *m*/*z* vs. migration time values) were detected. Fifty metabolites were identified based on *m*/*z*, migration time and/or MS/MS match to reference metabolome database. This figure demonstrates the separation of seven different metabolites. No signal was detected before ~11 min in any of the presented electropherograms.

The metabolite identifications were inspected. A total of 122 “metabolite-like” molecular features were measured in the CE-ESI-MS data. We identified 50 of these features (Table 1) by comparing our data against our CE-ESI-MS(/MS) metabolome database [29], developed using the same analytical platform used in this study. A total of 50 metabolites were identified through matching (aligning, Methods) the empirically obtained accurate mass-to-charge (*m*/*z*) values within 3 ppm and migration time within 3% acceptable error. Our approach to metabolite identification follows the recommendations of the International Chemical Analysis Working Group (CAWG) Metabolomics Standards Initiative (MSI), ensuring high fidelity and reproducibility [38]. The identifications are tabulated in Table 1. Figure 2 exemplifies the separation of seven of randomly selected metabolites, presented as narrow electrophoretic peaks. Their under-the-curve areas are a quantitative metric of concentration on our microanalytical high-sensitivity CE-ESI-MS platform, as validated elsewhere [30].

**Figure 3:**
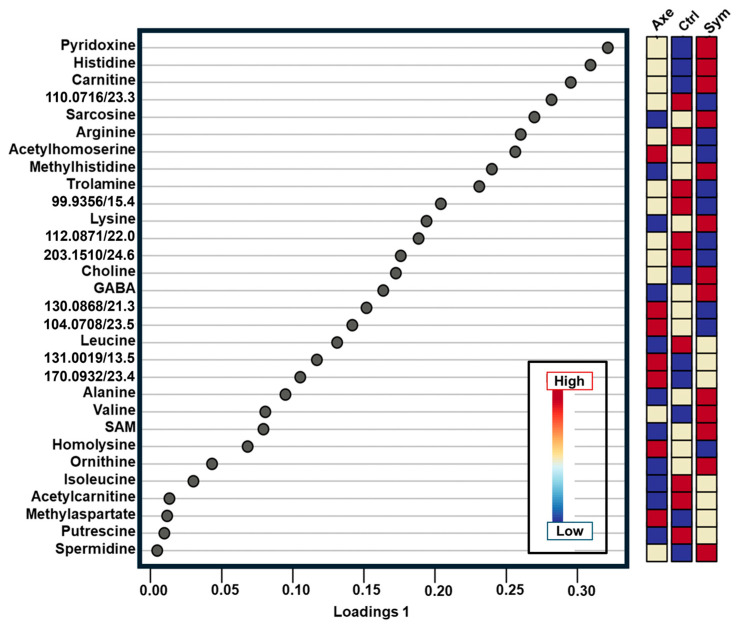
Multivariate statistical differentiation of the axenic (Axe), symbiotic (Sym) groups from the control (Ctrl). The 122 detected molecular features, signals with distinct *m*/*z* vs. migration time, MT (labelled *m*/*z*/MT pairs), were quantified. The supervised sparse least squares-discriminant analysis weighed the features’ discrimination ability. The top 30 most influential features are shown. The identified metabolites are labelled. For example, pyridoxine, histidine, and carnitine were highly enriched in the Sym nematode but depleted in the Ctrl. Key: GABA, γ-aminobutyric acid; SAM, S-adenosylmethionine.

Sparse partial least squares discriminant analysis (sPLS-DA) was recruited to extract molecular signatures among the known experimental groups. As shown in Figure 3, complex molecular profiles emerged during this supervised data analysis. Pyridoxine, histidine, and carnitine had elevated levels in the Sym group but were diminished in the Axe compared to Ctrl. Pyridoxine (vitamin B_6_) is among a group of vitamins (B_9_, B_12_, B_1_, and B_6_), which are dietary sources that are involved in biosynthetic processes such as purine and thymidylate synthesis and homocysteine re-methylation [39]. Histidine perturbations are reported to affect the nucleolar and cellular growth of clones in *D. melanogaster* [40], whereas L-Carnitine catalyzes the exchange of acyl groups between L-carnitine and coenzyme A (CoA), to form acylcarnitines [41]. No carnitine biosynthesis pathway has been reported for *D. melanogaster*, but there was a presumptive biosynthesis of L-carnitine in the fly due to the presence of putative orthologs of carnitine biosynthesis genes in the genome [42]. Therefore, we speculate that the exposure of *D. melanogaster* larvae to symbiotic *S. carpocapsae* nematodes carrying *X. nematophila* bacteria increases the production of energy metabolites and DNA biosynthesis, perhaps to cope with the infection from the nematode–bacteria symbiotic complex. Gamma–aminobutyric acid (GABA), sarcosine, trolamine, and lysine were enriched in the Sym group compared to the Axe. Seventy-two unidentified molecular features were also quantified as potential targets for more in-depth data analysis. These metabolite profiles offered a window into the metabolic pathway regulation sustaining the interaction between the *D. melanogaster* host and the nematode parasite.

We attempted to build a core metabolic pathway–activity profile considering the most differentially phenotypical features. The metabolites that were most differentially enriched among the groups were mapped against canonical metabolic pathways in *D. melanogaster*. These metabolites were searched against the Kyoto Encyclopedia of Genes and Genomes, KEGG (Methods). However, this model is still a rudimentary model of biology; for example, it was unable to consider chemical signalling, an emerging factor underpinning host–pathogen outcomes [43]. Table 2 tabulates the representative pathways to statistical significance (*p* < 0.05), with a pathway impact greater than zero marking pronounced differential enrichment. Pathways involving various amino acid biosynthesis (arginine, phenyl alanine, tyrosine, and tryptophan) and metabolism (arginine, proline, alanine, aspartate and glutamate, glycine, serine and threonine) were highly impacted (pathway impact greater than 0.5). Glutamate was reported to be a neurotransmitter in *D. melanogaster*; it was identified in all three groups in our measurements [44]. Arginine and proline metabolism was highly represented. Dietary L-arginine was reported to cause accelerated larval development and affect the total protein concentrations in third instar larvae [45]. This pathway is related to arginase, proline dehydrogenase (slgA), and delta [1]-pyrroline-5-carboxylate synthase (P5CS) genes in *D. melanogaster*. Overall, the results for these multiple pathways showcase the utility of high-sensitivity CE-ESI-MS metabolomics profiling in investigating host–pathogen interactions. The metabolome data, in turn, can help guide experiments to identify molecules specific to pathogenic exposure and determine their biological functions.

## 3. Conclusions

In this study, we performed untargeted metabolomics profiling on extracts from *D. melanogaster* larvae infected with Sym *S. carpocapsae* (nematodes containing the mutualistic bacteria *X. nematophila*) or Axe *S. carpocapsae* (nematodes lacking *X. nematophila* bacteria) using high-sensitivity microanalytical CE-ESI-MS. To the best of our knowledge, this is the first report of metabolomics in *D. melanogaster* in the context of entomopathogenic nematode infection when encountering Sym and Axe parasites. We detected 122 molecular features across the three sample groups and identified ~50 metabolites with high analytical confidence. Relative quantitative profiling of the 122 compounds found systematic metabolic differences among the host–infection groups. Pyridoxine, histidine, carnitine, sarcosine, methylhistidine, choline, γ-aminobutyrate, and lysine were highly enriched in the symbiotic-nematode-infected larvae but diminished in the axenic-nematode-infected individuals. In contrast, acetylhomoserine, homolysine, and methylaspartate were enriched in the axenic group. Of note, *D. melanogaster* wild-type larvae succumbed to infection by *S. carpocapsae* symbiotic and axenic nematodes at a similar rate [43]. Additional research is needed to determine whether the production of the detected metabolites plays a protective or detrimental role in entomopathogenic nematode infection. Further studies will explore the role of the detected metabolites, their functions and interactions with entomopathogenic nematode-excreted/secreted products, the relevant pathways that regulate these processes, and the host immune or stress mechanisms which are activated or circumvented. This information will reveal the mechanistic basis of entomopathogenic nematode parasitism in *D. melanogaster* and perhaps in other insects. Future work will also test the metabolite changes induced in *D. melanogaster* larvae and adult flies in response to injection with the mutualistic bacteria *Photorhabdus* spp. and *Xenorhabdus* spp., which are found in the entomopathogenic nematodes *Heterorhabditis* spp. and *Steinernema* spp., respectively. This work demonstrates a novel bioanalytical ability when using CE-ESI-MS to profile metabolites in the *D. melanogaster* model during infection with other widely used entomopathogenic nematodes, such as *Heterorhabditis bacteriophora* [46]. The CE-ESI-MS approach is also adaptable to other biological model organisms, sharpening the analytical toolset of immunology and host–pathogen interaction studies.

## 4. Materials and Methods

**Materials and Reagents:** Reagent-grade acetic acid (AcOH), formic acid (FA), and LC-MS-grade water and methanol (MeOH) were purchased from Fisher Scientific (Pittsburgh, PA, USA). Acetylcholine was acquired from Acros Organics (now Thermo Scientific Chemicals, Waltham, MA, USA). The fused silica capillaries (40/105 μm inner/outer diameter) for CE were obtained from Polymicro Technologies (Phoenix, AZ, USA) and used without chemical modification. The CE-ESI emitter was built from a 1″ long segment of stainless-steel hypodermic tubing (165/210 µm inner/outer diameter, part no. HTX-33X, Small Parts, Inc., Miramar, FL, USA).

**Solvents and Standards:** The metabolite extraction solvent was prepared to contain 0.5% (*v*/*v*) AcOH in 50% (*v*/*v*) water/MeOH. The CE-ESI sheath solution comprised 0.1% (*v*/*v*) FA in 50% (*v*/*v*) water/MeOH. The CE background electrolyte (BGE) was 1% FA in LC-MS-grade water.

**Fly Strains, Nematodes, and Injection Experiments:** The *D. melanogaster* line Oregon-R was used in all the experiments. All flies were grown on *Drosophila* media (Meidi Laboratories) and approximately 10 granules of dry baker’s yeast. All fly stocks were maintained at 25 °C with a 12 h light/12 h dark cycle. Late second-early to third instar larvae were used for all experiments. The entomopathogenic nematode *S. carpocapsae* carrying the mutualistic bacteria *X. nematophila* (Sym) were reared in the larvae of the greater wax moth *Galleria mellonella*. Axe *S. carpocapsae* nematodes were cultured using an established protocol [36]. *S. carpocapsae* carrying the mutualistic bacteria *X. nematophila* were washed with 1% bleach, followed by five rinses with water to remove any traces of bacteria or bleach to generate the Axe nematodes. Infective juveniles between 2 and 3 w of age were selected for all infection experiments.

**Generation of axenic nematodes:** The mutant bacteria *X. nematophila* Δ*rpoS* were used for generating *S. carpocapsae* axenic nematodes. For bacterial inoculation, the bacteria were supplemented with 50 μg/mL Ampicillin and 30 μg/mL Kanamycin and grown in 2 mL Luria–Bertani (LB) broth overnight at 30 °C in a shaker–incubator at 220 rpm. A total of 250 μL of the overnight culture was added to fresh 5 mL LB and the mix was incubated on a shaker at 30 °C for 22-to-24 h.

To prepare oily agar plates, 300 mL of growth media containing 2.4 g of nutrient broth, 4.5 g of bacteriological agar, and 1.5 g of yeast extract was added to 267 mL of distilled water. The mix was autoclaved, and the following components were then added to the media: 3 mL of 0.98 M MgCl_2_, 28.8 mL of 7.3% sterile corn syrup, and 1.2 mL of sterile corn oil. After autoclaving the solution, the antibiotics were added to the media and the mix was stirred and then poured into one side of the biplates. The *X. nematophila* Δ*rpoS* bacterial culture (100 μL) was pipetted onto the oily agar media and spread evenly with a sterile spreader. The plates were incubated at 30 °C for 24 h.

For nematode sterilization, high-density *S. carpocapsae* nematodes were resuspended in 1 mL of sterile water and then pipetted into a 1.5 mL Eppendorf tube. The nematodes were pelleted via 10 s centrifugation to 13,000 rpm at room temperature. The supernatant was discarded, and 1 mL of freshly prepared 1% bleach solution was added to the nematode pellet. The suspension was mixed well and centrifuged, and the supernatant was discarded. The process was repeated. The nematode pellet was washed in 1 mL of sterile distilled water to remove the bleach residue. The washing step was repeated 5 times. The pellet was resuspended in 100 μL distilled water. The nematode concentration was counted under a stereoscope.

About 500 to 700 surface-sterilized nematodes were transferred to the bacterial plates incubated on moist paper towels for ~10 d. The plates were observed under a stereoscope to monitor the age and condition of the nematodes. When the infective juvenile stage was reached, in approximately 2 to 3 weeks, water traps were prepared, and the nematodes were collected in cell culture flasks. This process was repeated twice to ensure a ~100% Axe population. To establish axenicity, a high-density pellet of the collected nematodes was surface-sterilized and homogenized in a 1.5 mL Eppendorf tube. This was then plated on LB-Agar plates. Absence of any bacterial growth on the plates indicated that the nematodes were axenic.

**Entomopathogenic nematode infections:** To infect *D. melanogaster* larvae with *S. carpocapsae* nematodes, 100 μL of 1.5% agarose gel was added to the wells of a 96-well microtiter plate. The agarose was allowed to cool for 3 h. Third instar *D. melanogaster* larvae (Oregon strain) were transferred onto a Whatman filter paper using a fine soft bristle paintbrush. To remove any food debris, they were washed via pipetting a small drop of sterile water. A drop of 10 μL of sterile distilled water containing 100 Sym or Axe surface-sterilized *S. carpocapsae* nematodes and a single *D. melanogaster* larva were added to each microtiter plate well. Each row of the 96-well plate was covered with a strip of PCR clear film (Eppendorf), and holes were poked to allow for air circulation. Infected and uninfected control *D. melanogaster* larvae were frozen at 78 h after infection with the nematode parasites.

**Metabolite Extraction:** The samples consisted of Sym (n = 4), Axe (n = 3), and Ctrl (n = 4) biological replicates. Metabolites from each experimental condition were extracted in 250 µL of 50% MeOH containing 0.5% (*v*/*v*) AcOH, facilitated via periodical sonication in an ice-cold water bath. This protocol was selected based on previous studies establishing the efficiency and reproducibility of extraction for CE-ESI-MS experiments [28]. After ~30 min of treatment, the larvae appeared homogenized to the eye, which was considered the extraction endpoint in this study. The samples were vacuum-dried at 4 °C to enhance chemical stability. The resulting metabolite extracts, henceforth called the “samples”, were reconstituted in 7 μL of extraction solvent and stored at –80 °C until analysis.

**CE-ESI-MS Measurement**: A 500 nL portion of each extract was deposited into a sample-loading microvial. A ~10 nL portion was hydrodynamically loaded into a fused silica capillary following an established protocol [27] and analyzed using a custom-built CE-ESI platform validated elsewhere [30,37]. The samples were electrophoresed in the BGE-filled fused silica separation capillary (40/105 μm inner/outer diameter) through electrifying its inlet end to +19.5 kV (vs. Earth ground), drawing an ~8 µA current. As analytes migrated toward the outlet end of the capillary, they were fed online into co-axial blunt-tip-emitter CE-ESI interface built and operated as reported elsewhere [26,27,28]. At a distance of ~3 mm and –1700 V spray potential, microscopy monitoring revealed the 1 μL/min sheath flow to channel into a robust Taylor cone, ensuring high ionization efficacy via the ensuing cone-jet regime [47]. A quadrupole orthogonal accelerated time-of-flight mass spectrometer (Impact HD, Bruker Daltonics, Billerica, MA, USA) was used to analyze the cations generated between *m*/*z* 50 and 500 at 40,000 full-width-at-half-maximum resolution. The instrument was sensitivity-tuned, *m*/*z*-calibrated, and operated following the vendor’s protocols and software packages.

**Data Analysis.** The MS files were analyzed using Compass Data Analysis 4.3 (Bruker Daltonics). Each primary (raw) data point was internally *m*/*z*-calibrated on sodium–formate clusters that formed in the ion source [30]. The molecular features (signals with unique *m*/*z* vs. migration time information) were extracted with ± 5 mDa *m*/*z* accuracy using our semi-automatic script [27]. As validated elsewhere [27], we used a third-order polynomial to align the measured molecular profiles to our in-house-developed CE-ESI-MS metabolome database [29] on the same analytical platform as employed here. The relative concentration of each *m*/*z*–MT-specific molecular feature was estimated based on its under-the-curve peak area, as validated elsewhere on the same instrumental platform used here [30]. These data depicted the metabolome through the 122 metabolite features monitored here, including the 50 identified features (Table 1).

The quantitative metabolite data from the three sample groups were normalized to the median abundance, log_10_-transformed, and data-scaled for sPLS-DA in MetaboAnalyst 6.0. sPLS-DA was proven to consider the broad replicate conditions for the features driving the known differences [48]. We employed 10-fold cross-validation to extract the components that yielded the lowest error in phenotype discrimination. The Kyoto Encyclopedia of Genes and Genomes (KEGG) metabolomic database [49] enabled the metabolic pathway analyses with the following settings: pathway library, *Drosophila melanogaster* (fruit fly); pathway algorithm, overrepresentation via hypergeometric test; topology via relative betweenness centrality. The quantitative metadata were plotted in OriginPro 2022 9.9.0.225 (OriginLab, Northampton, MA, USA).

**Study Design, Rigour, and Statistics:** Measurements were taken using n = 4 biological replicates for the control and symbiotic groups and n = 3 for the axenic group. At the start of each day of CE-ESI-MS analysis, the platform was validated on 300 amol of acetylcholine based on the observed sensitivity, *m*/*z* accuracy, and reproducibility. The following criteria provided robustness and reproducibility based on our accumulated experience with the technology: identification fidelity of <5% relative standard deviation (RSD) in corrected MT, <25% relative standard deviation (RSD) in the quantified signal abundance (under-the-curve peak area in *m*/*z*-extracted ion electropherograms), and signal-to-noise ratio (S/N) > 15.

**Safety Considerations:** Standard laboratory safety measures were taken throughout the experiments. CE-ESI emitters present a puncture hazard; extra care was taken while handling them. The connective parts of the CE setup were enclosed in a safety interlock-enabled system or earth-grounded to prevent electrical shock hazard.

## Figures and Tables

**Table 1:** List of molecular features, mass-to-charge (*m*/*z*) vs. migration time signals, matched in the control, axenic, and symbiotic nematode-infected *Drosophila melanogaster* against an in-house-developed CE-ESI-MS metabolome database. Key: Δ, deviation from the reference (% or ppm).

No.	Metabolite	Migration Time		*m*/*z*		
		(min)	Δ (%)	Measured	Theoretical	Δ (ppm)
1	Spermidine *^,^**	8.5	1.6	146.1648	146.1652	0.4
2	Putrescine *	9.1	−0.9	89.1072	89.1073	0.1
3	Choline **	15.3	−1.8	104.1068	104.1070	0.2
4	S-adenosylmethionine *^,^**	16.3	−2.7	399.1447	399.1445	−0.2
5	Ornithine *^,^**	16.4	−1.3	133.0973	133.0972	−0.1
6	Sarcosine *	16.6	−1.6	90.0550	90.0550	0.0
7	Lysine *^,^**	16.6	−1.5	147.1128	147.1128	0.0
8	Arginine *^,^**	17.2	−1.2	175.1190	175.1190	0.0
9	Homolysine *^,^**	17.3	−1.1	161.1286	161.1285	−0.1
10	γ-aminobutyrate *^,^**	17.4	−0.9	104.0703	104.0706	0.3
11	Histidine **	17.4	−0.8	156.0769	156.0768	−0.1
12	N_6_,N_6_,N_6_-trimethyllysine *^,^**	17.8	−2.6	189.1607	189.1598	−0.9
13	Methylhistidine *^,^**	18.3	−2.3	170.0924	170.0924	0.0
14	Guanidinopropanoate *^,^**	18.5	−1.9	132.074	132.0768	2.8
15	Acetylcholine *^,^**	18.7	−1.6	146.1175	146.1176	0.1
16	Guanine *^,^**	18.7	2.1	152.0570	152.0567	−0.3
17	Trolamine *	18.8	−0.9	150.1126	150.1125	−0.1
18	Carnitine *^,^**	20.5	−3.9	162.1127	162.1125	−0.2
19	Pyridoxine (Vitamin B_6_) *^,^**	20.9	−1.3	168.0660	168.0655	−2.4
20	Acetylcarnitine *^,^**	22.3	−1.8	204.1233	204.1230	−0.3
21	Methylaspartate *^,^**	22.3	1.8	148.0606	148.0604	−0.2
22	Propionylcarnitine *^,^**	23.1	−2.3	218.1399	218.1387	−1.2
23	Glycine *	23.2	−1.7	76.0395	76.0393	−0.2
24	Creatine *^,^**	23.6	−2.4	132.0771	132.0768	−0.3
25	Adenosine *	24.7	−2.4	268.1047	268.1040	−0.7
26	Alanine *	25.5	0.6	90.0553	90.0550	−0.3
27	Argininosuccinate *^,^**	27.4	−0.6	291.1305	291.1299	−0.6
28	Valine *^,^**	29.9	1.1	118.0863	118.0863	0.0
29	Serine *^,^**	30.4	−0.2	106.0498	106.0499	0.1
30	Isoleucine *	30.2	1.2	132.1020	132.1019	−0.1
31	Leucine *	30.5	1.2	132.1020	132.1019	−0.1
32	Threonine *^,^**	32.5	0.6	120.0654	120.0655	0.1
33	Asparagine *^,^**	32.6	0.1	133.0610	133.0608	−0.2
34	Tryptophan *^,^**	33.2	0.5	205.0975	205.0972	−0.3
35	Methionine *^,^**	33.4	0.7	150.0586	150.0583	−0.3
36	Glutamine *^,^**	33.5	0.6	147.0767	147.0764	−0.3
37	Acetylhomoserine *	34.1	0.4	162.0751	162.0761	1.0
38	Citrulline *^,^**	34.1	0.2	176.1032	176.1030	−0.2
39	Homocitrulline *^,^**	34.1	0.5	190.1191	190.1186	−0.5
40	Glutamate *^,^**	34.2	0.5	148.0607	148.0604	−0.3
41	Phenylalanine *^,^**	34.9	1.1	166.0867	166.0863	−0.4
42	Acetyllysine *	35.3	1.2	189.1237	189.1234	−0.3
43	Tyrosine *^,^**	35.4	1.3	182.0817	182.0812	−0.5
44	Proline *^,^**	36.6	0.6	116.0706	116.0706	0.0
45	Hypoxanthine *^,^**	36.7	−0.8	137.0458	137.0458	0.0
46	Cysteine *^,^**	37.1	5.0	122.0270	122.027	0.0
47	Aspartate *^,^**	39.2	1.5	134.0447	134.0448	0.1
48	Glycine betaine *	40.0	1.4	118.0862	118.0863	0.1
49	Hydroxyproline *^,^**	43.3	1.0	132.0657	132.0655	−0.2
50	Glutathione *^,^**	46.7	0.1	308.0924	308.0911	−1.3

**Note:** Asterisk (*) indicates matching accurate *m*/*z* and migration time with an in-house-developed CE-MS metabolome database (see text). Double asterisk (**) represents identifications supported by MS/MS in the database.

**Table 2:** KEGG statistical pathway analysis for the 30 most differentially enriched metabolites between the control and symbiotic nematode infections. Statistical significance was marked at *p* < 0.05 and a pathway impact greater than zero.

Pathway	Total	Hits	*p*-Value	Impact
**Symbiotic group**				
Arginine and proline metabolism	29	4	1.91 × 10^−4^	0.333
Alanine, aspartate, and glutamate metabolism	23	2	0.0260	0.122
One carbon pool via folate	23	2	0.0260	0.168
Glutathione metabolism	26	2	0.0327	0.0179
Vitamin B6 metabolism	8	1	0.0872	0.0833
Histidine metabolism	9	1	0.0976	0.400
Arginine biosynthesis	13	1	0.138	0.146
Butanoate metabolism	14	1	0.148	0.200
Glycerophospholipid metabolism	32	1	0.309	0.0542
Cysteine and methionine metabolism	32	1	0.309	0.0546
**Control group**				
Arginine and proline metabolism	29	2	0.00418	0.271
Arginine biosynthesis	13	1	0.0481	0.341
Glutathione metabolism	26	1	0.0945	0.0179

## Data Availability

The data supporting the findings of this study are available within the article.

## Abbreviations

The following abbreviations are used in this manuscript:

Ctrl	Control
Axe	Axenic
BGE	Background electrolyte
CE-ESI-MS	Capillary electrophoresis electrospray ionization mass spectrometry
FA	Formic acid
GABA	Gamma–aminobutyrate
GC	Gas chromatography
KEGG	Kyoto encyclopedia of genes and genomes
LC	Liquid chromatography
MS	Mass spectrometry
MT	Migration time
sPLS-DA	Sparse least squares–discriminant analysis
Sym	Symbiotic
MS/MS	Tandem mass spectrometry
